# Circulatory GSK‐3b; A blood based biomarker and a therapeutic target for Alzheimer's Disease

**DOI:** 10.1002/alz70856_106304

**Published:** 2026-01-08

**Authors:** Sakshi Kumari, Sharmistha Dey

**Affiliations:** ^1^ All India Institute of Medical Sciences, New Delhi, Delhi, India

## Abstract

**Background:**

Alzheimer's disease (AD) is the progressive neurological disorder, which causes degeneration of neuronal cells and memory loss. The neuropathological presentation of AD constitute amyloid beta (Aβ) plaques and hyperphosphorylated tau tangles. Hyperactivation of GSK‐3b and p53 results in the formation of these neuropathological hallmarks. In this study, we have explored the potential of GSK‐3b and p53 as blood‐based biomarkers for early detection of AD.

**Method:**

The circulatory level of GSK‐3b, phospho‐GSK‐3b, p53 and phospho‐p53 in serum from AD, mild cognitive impairment (MCI), and geriatric‐control (GC) subjects were measured using surface plasmon resonance and further validated by western blot. The rescue of neurotoxicity by an antioxidant plant extract, Emblica Officinalis (EO) was also checked by MTT assay on SH‐SY5Y cells (neuroblastoma cell line).

**Result:**

The level of all the proteins were found to be significantly upregulated (*p* > 0.001) in AD and MCI compared to GC and can easily differentiate AD and MCI from GC. The expression of these proteins was found to be decreased in a dose dependent manner when treated with EO in SH‐SY5Y cells.

**Conclusion:**

These proteins can serve as potential blood based biomarkers for the diagnosis of AD and EO can suppress their level. This work has translational value and clinical utility in the future.

